# Retrospective study of preterm infants exposed to inhaled nitric oxide in Kaiser Permanente Southern California: morbidity, mortality and follow-up

**DOI:** 10.1038/s41372-024-02051-w

**Published:** 2024-07-18

**Authors:** Dilip R. Bhatt, David Braun, Roman Angelo Dizon, Jiaxiao M. Shi, Sunjeeve Weerasinghe, Alex Sabio, Siva Reddy, Henry C. Lee, Rangasamy Ramanathan, Satyan Lakshminrusimha

**Affiliations:** 1 Fontana Medical Center, Kaiser Permanente Southern California, Fontana, CA USA; 2 Department of Research and Evaluation, Kaiser Permanente Southern California, Pasadena, CA USA; 3 University of California, San Diego, CA USA; 4 Division of Neonatology, Cedars Sinai Guerin Children’s, Cedars Sinai Medical Center, Los Angeles, CA USA; 5 UC Davis Children’s Hospital, Sacramento, CA USA

**Keywords:** Paediatrics, Respiratory tract diseases, Outcomes research

## Abstract

**Objective:**

Describe characteristics of preterm infants exposed to inhaled nitric oxide (iNO) in Kaiser Permanente Southern California.

**Study design:**

Case review of preterm infants <34-weeks exposed to iNO during 2010–2020 including respiratory and echocardiographic status, NICU course, and 12-month follow-up.

**Results:**

270 infants, 2.63% of births<34 weeks, (median, range: 26.1, 22^5/7^–33^6/7^ weeks gestation) were exposed to iNO. Median FiO_2_ at iNO initiation was 1.0 (IQR 0.94-1.0). Pulmonary hypertension (PH) was not associated with risk-adjusted 2 h oxygenation response or improved survival. Mortality to NICU discharge was 37.4%. Median cost of iNO was $7,695/patient. Discharged survivors experienced frequent rehospitalization (34.9%), use of supplemental oxygen, sildenafil, diuretics, bronchodilators, and steroids. Four infants had persistent PH. Five infants died after NICU discharge.

**Conclusions:**

Preterm infants receiving iNO have high mortality and 1st year morbidity. As currently used, iNO may be an indicator of respiratory disease severity rather than mediator of improved outcomes.

## Introduction

Inhaled nitric oxide (iNO) is the only FDA-approved pulmonary vasodilator in neonates. It is beneficial in decreasing the need for extracorporeal membrane oxygenation (ECMO) in late preterm and term infants greater than 34 weeks’ gestation at birth [1]. Consensus statements from the National Institutes of Health (NIH) [2] and a clinical report from the American Academy of Pediatrics (AAP) [3] do not recommend iNO use in preterm infants. Despite these recommendations, the use of iNO in preterm infants is common in both academic and non-academic settings such as the NICHD Neonatal Research Network, Canadian Neonatal Network, and Pediatrix® institutions [4–6]. The safety profile of iNO, improvement in oxygenation [7–9], lack of alternative therapies, and an inherent urge for patient-centered care have led to continued use of iNO in preterm infants [10]. Preterm infants with pulmonary hypoplasia [11, 12], prolonged rupture of membranes (PROM) and echocardiographic evidence of pulmonary hypertension (PH) are thought to be more responsive to iNO [12–14]. Selective use of iNO in preterm infants with PH physiology or pulmonary hypoplasia is considered a pragmatic approach [15]. These retrospective database studies included large number of preterm infants exposed to iNO but provided only limited information on clinical condition or echocardiographic findings at initiation of iNO, response to iNO, or follow-up data. Also attempts to compare iNO-exposed infants to non-iNO exposed control groups retrospectively were hampered by the many important latent variables, such as center variations, ventilator practices or criteria for starting iNO, which are difficult to determine retrospectively.

Kaiser Permanente South California Health system (KPSC), a 4.7 million member integrated health care system [16] provides a large population-based birth cohort to describe the clinical characteristics of preterm infants receiving iNO and assess for evidence of iNO effects. To avoid the methodological challenges of creating a meaningful non-iNO control group, we limited our assessment of iNO effects to the comparison of subpopulations with or without risk factors known to alter the response to iNO.

The specific objectives of this study were: (1) to identify all preterm infants <34 weeks gestation born in KPSC between 2010 and 2020 who received iNO, (2) provide a detailed description, based on individual chart review, of their clinical course including their condition and echocardiographic findings at initiation of iNO, their response to iNO, their NICU outcomes and their respiratory course during infancy, (3) to test the hypothesis that preterm infants with echocardiographic PH at the time of iNO initiation in the first 10 days of life will have better initial oxygenation response and survival compared to infants without evidence of PH, and (4) to test the hypothesis that infants with PROM would have better survival than those without PROM.

## Methods

We conducted a retrospective study of all preterm infants admitted to KPSC NICUs at <34 weeks gestation between Jan 1, 2010, and December 31, 2020, who received iNO therapy during their NICU course. The study period was chosen to include all years after implementation of the KPSC electronic medical record. Seven KPSC centers provide iNO and patients from other centers deemed to require iNO were transported to these centers for care. There were no formal guidelines in KPSC for iNO use in the NICU. Neonatologists do not need administrative approval to initiate iNO for treatment of hypoxemic respiratory failure (HRF) or PH. During this period, iNO was not used for prophylaxis for bronchopulmonary dysplasia (BPD) within the network.

The study was approved by the KPSC institutional review board. Data was obtained by review of the electronic health record. Race and ethnicity were based on self-report by mothers. Exposure to iNO therapy was identified by documentation in hospitalizations submitted to Vermont Oxford Network (VON) registry [17] via the California Perinatal Quality Collaborative registry [18, 19] in which all KPSC hospitals and over 90% of all California NICUs participate [20]. The VON definition of BPD (supplemental oxygen at 36 weeks corrected age or discharge home on supplemental oxygen if discharged before 36 weeks corrected age) was used for analysis. Data for BPD as defined by Jensen et al was also analyzed [21]. Chart review using echocardiographic criteria outlined by Weismann et al were used to diagnose PH [22].

Oxygenation response to iNO was arbitrarily defined as an absolute decrease of 25% in inspired oxygen concentration with an increase in SpOFiO_2_ (S/F) ratio by 30 or greater within 2 h of iNO initiation.

Mortality was also studied among the subgroup of infants echocardiographically classified as PH or no PH in association with iNO initiation in the first 10 days of life were compared.

Costs were determined by applying the contracted nitric oxide cost structure to the nitric oxide exposures across the study population factored by the duration and year of use. All infants followed up in the KPSC system were included in the analysis.

### Statistical analysis

We assessed differences in the distribution of categorical variables using the chi-square test and continuous variables using two-sided t test. Subgroups based on PROM, pulmonary hypoplasia, echocardiographic evidence of PH, race, and ethnicity were analyzed by chi-square test. Covariates used for risk-adjustment of odds ratio (OR) included year of birth, gestational age, weight for gestational age (appropriate for gestation-AGA, small for gestation-SGA, and large for gestation-LGA) status, facility and sex were determined. Statistical significance was set at *p* < 0.05. Analysis was performed with SAS statistical software version 9.4 (SAS institute, Cary NC).

## Results

A total of 418206 infants were born within KPSC hospitals from 2010 through 2020 including 10252 babies <34 weeks gestation (2.54%). Among all births, 750 infants (0.18%) were exposed to iNO in the NICUs during this period. Thirty-six percent of all infants exposed to iNO (270) were <34 weeks gestational age. The incidence of iNO exposure was 2.63% among preterm births <34 weeks. The rate of iNO exposure reported in the California Perinatal Quality Care Collaborative during the same period [17, 18]. was 2.39% 2539/107876. Two-thirds of these infants (178) were <29 weeks gestation. All infants exposed to iNO irrespective of congenital anomalies, chromosomal aneuploidy, or moribund status were included in the analysis.

### Baseline characteristics

**Fig. 1:**
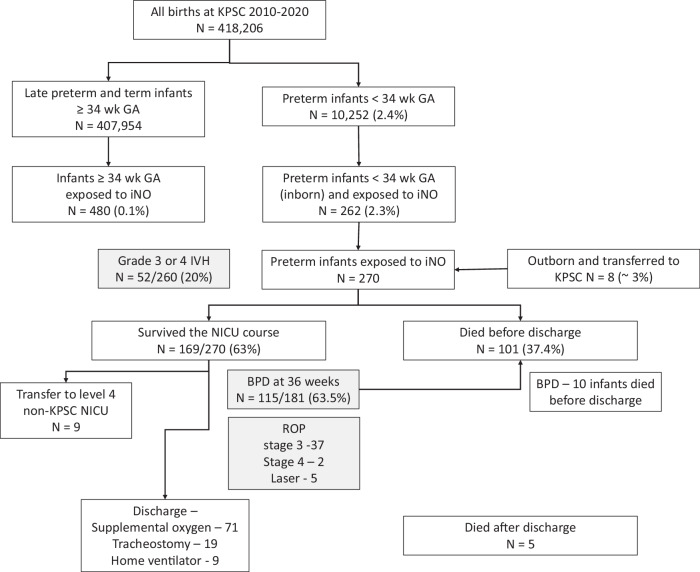
Flowchart of newborn infants born at KPSC between 2010 to 2020. Preterm infants <34 weeks gestation exposed to iNO (*n* = 270), and their outcomes are shown. GA gestational age, IVH intraventricular hemorrhage, BPD bronchopulmonary dysplasia, ROP retinopathy of prematurity.

Baseline characteristics of these infants are shown in Table 1 and the flowchart is shown in Fig. 1. Eight infants were born outside the Kaiser network and transferred to KPSC institutions. One growth restricted infant was born at 26 weeks of gestation with a birth weight of 270 g and developed HRF following ductal ligation and received iNO and survived.

**Table 1:** Baseline characteristics of preterm infants (<34 weeks GA) exposed to iNO (*n* = 270).

Characteristics	*N* or mean or median
Inborn (%)	262 (97%)
Delivery by cesarean section	200 (74.1%)
Male	154 (57%)
Gestational age in weeks (median, range)	26 (22^5/7^–33^5/7^)
Birth weight in grams (median, range)	783 (270–3200)
Small for gestational age (SGA)	37 (13.7%)
Large for gestational age (LGA)	21 (7.8%)
Ethnicity and Race (by maternal self-report)	
Hispanic (%)	128 (47.4%)
White (%)	62 (23.0%)
Black or African American (%)	49 (18.1%)
Asian (%)	24 (8.9%)
Hawaiian (%)	3 (1.1%)
Apgar score – 1 min (median, range)	4 (0–9)
Apgar score – 5 min (median, range)	7 (1–9)

### Initiation of iNO

Characteristics of infants at the time of initiation of iNO and duration of iNO exposure are shown in Table 2. The indications for iNO were HRF and PH. Majority of patients (237/270–87.8%) were in HRF and required inspired oxygen >70%. Three-fourth of infants were on 100% inspired oxygen at initiation of iNO. Fifteen infants (5.6%) were receiving ≤50% oxygen and had clinical or echocardiographic evidence of PH (3 infants on 21–30% oxygen, 3 on 31–40% and 9 on 41–50%). Fifty-nine percent of patients were acidotic with a blood pH < 7.25 at iNO initiation. Thirty-two infants (12%) had pH < 7.0 at initiation of iNO. Thirty-five infants 14.6% of 240 infants with sepsis workups had a positive blood culture at the time of iNO initiation.

**Table 2:** Characteristics at initiation and duration of iNO exposure (*n* = 270 unless specified).

Characteristic	*N* or mean or median	% Or SD or IQR
Age at iNO initiation (h) median IQR	54	(11–457)
<8 Days	157	58.1%
8–28 Days	75	27.8%
>28 Days	38	14.1%
Pre-INO respiratory support		
Surfactant	263	97.4%
Non-invasive positive pressure ventilation	1	0.4%
Invasive mechanical ventilation	19	7.0%
High frequency oscillator	239	88.5%
High frequency jet ventilator	7	2.6%
PPV (bagging or T-piece)	4	1.5%
Pre-iNO OI^a^ (data from 176 infants with arterial access)	30 (IQR – 20)	
Mild HRF OI ≤ 15 (%)	25	14.2%
Moderate HRF OI 16–25 (%)	39	22.2%
Severe HRF OI 26–40 (%)	66	37.5%
Critical HRF OI > 40 (%)	46	26.1%
Pre-iNO OSI^b^ (median – IQR)	17	14–21
Echocardiogram pre-INO or immediately after iNO	215	79.6%
Pulmonary hypertension on echocardiogram	86/215	40.0%
iNO dose, initial (ppm) – median and range	20	5–40
iNO dose, initial (5 ppm)	13	4.8%
iNO dose, initial (10 ppm)	29	10.7%
iNO dose, initial (15 ppm)	4	1.5%
iNO dose, initial (20 ppm)	223	82.6%
iNO dose, initial (40 ppm)	1	0.4%
iNO duration (h) median IQR	76	35–135
iNO duration 1–8 h (%)	27	10.0%
iNO duration 9–24 h (%)	21	7.8%
iNO duration 25–50 h (%)	52	19.3%
iNO duration 51–100 h (%)	75	27.8%
iNO duration 101–200 h (%)	58	21.5%
iNO duration 201–300 h (%)	18	6.7%
iNO duration 301–3000 h (%)	19	7.1%

^a^OI – oxygenation index = mean airway pressure (cm H_2_O)*FiO_2_*100 ÷ PaO_2_ (mmHg).

^b^OSI – oxygen saturation index = mean airway pressure (cm H_2_O)*FiO_2_*100 ÷ SpO_2_ (%).

### Oxygenation response to iNO

We arbitrarily defined oxygenation response to iNO as an absolute decrease of 25% in inspired oxygen concentration with an increase in SpO_2_/FiO_2_ (S/F) ratio by 30 or greater within 2 h of iNO initiation. 88 infants (88/270 = 32.6%) had a positive oxygenation response to iNO. Responders had lower SpO_2_ (74 ± 20% vs. 80 ± 13%) prior to initiation of iNO. Responders had a lower postnatal age (166 ± 305 vs. 385 ± 603 h) and higher incidence of PH on echocardiogram (49% vs. 35%) compared to non-responders. Survival to discharge was 72% among responders and 58% among non-responders but when corrected for gestational age and sex, this difference was not statistically significant.

### Echocardiography

Among the 84 patients who had echocardiograms performed prior to initiation of iNO, 36 (43%) had evidence of PH. However, only 58 echocardiograms were performed within 24 h of starting iNO and 33 (57%) had evidence of PH. Echocardiography was performed shortly after (usually on a subsequent working day) initiation of iNO in 131 (48.5%) and 50 of them (38%) had evidence of PH. Although most echocardiograms were performed during day-time hours, initiation of iNO occurred round-the-clock suggesting that factors other than echocardiographic evidence of PH (e.g., inability to maintain SpO_2_ despite 1.0 FiO_2_) were the primary drivers for initiation of iNO. Fifty-five patients who received iNO never had an echocardiogram done during the NICU course and 32 of these infants died (18 within 24 h of iNO initiation).

**Fig. 2:**
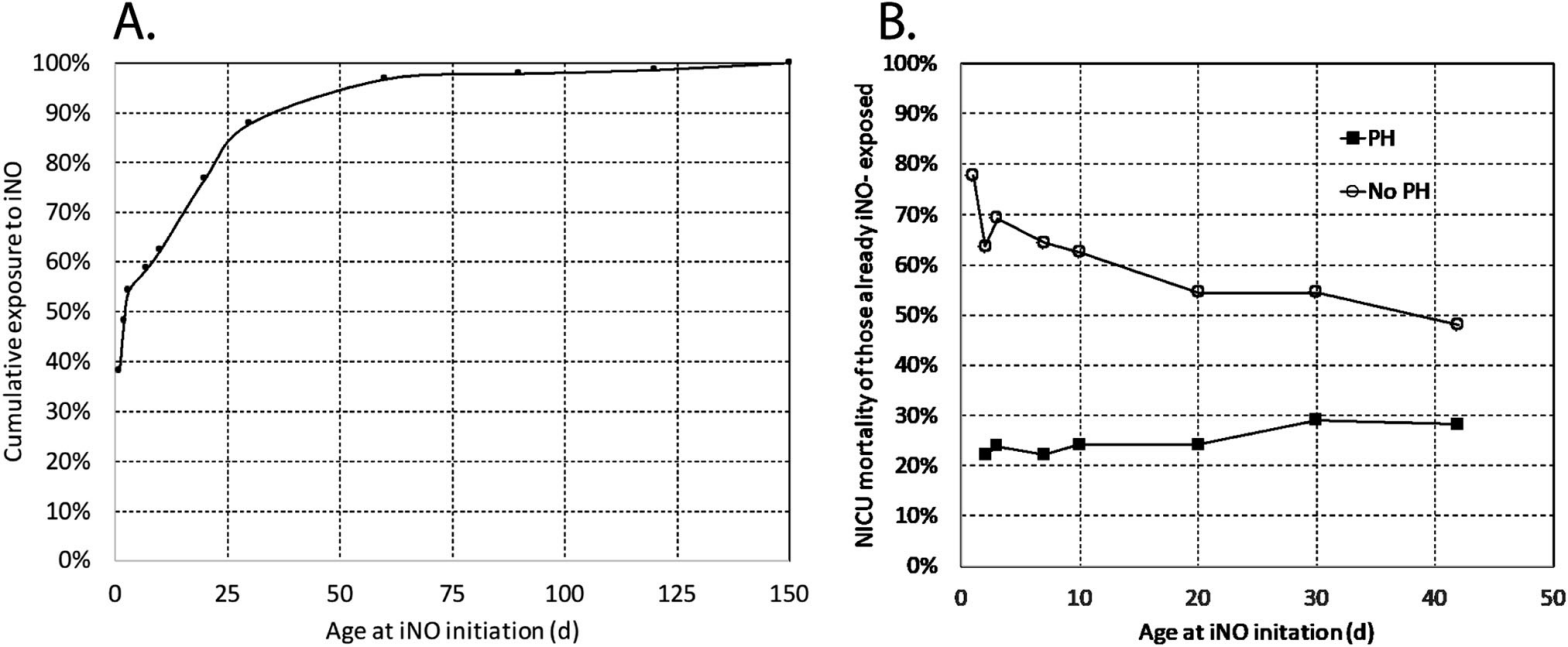
Day of iNO initiation, cumulative iNO exposure and cumulative mortality. **A** Cumulative percentage of preterm infants exposed to iNO based on day of initiation of iNO (*n* = 270). **B** Cumulative mortality among 58 infants with echocardiogram performed within 24 h prior to initiation of iNO. PH pulmonary hypertension.

Age at initiation of iNO had a wide distribution ranging from 1 h to 6.5 months (Fig. 2A) – majority of infants received iNO during the first week of postnatal period (58.1%) and some received iNO for PH associated with BPD after the first postnatal month (14.1%). Most infants were treated with surfactant (97.4%) prior to iNO and were ventilated with high frequency-oscillatory or jet ventilation (91.1%; Table 2).

*Starting dose* of iNO was 20 ppm in 223 (82.6%) of patients with a small number receiving either 5 ppm (4.8%), 10 ppm (10.7%) or 15 ppm (1.5%). One patient was started with 40 ppm of iNO.

### Duration of iNO therapy

The duration of iNO therapy ranged from 1 h to 107 days (Table 2). Thirty-five infants received multiple courses of iNO (26–2 courses, 7–3 courses and 2–4 courses). A third of patients (89/270) were exposed to >5 days of iNO therapy.

### Co-morbidities

At 36 weeks postmenstrual age, among 181 survivors, 115 infants (63.5%) met VON criteria for BPD. Ten infants with BPD at 36 weeks postmenstrual age died before NICU discharge. Intraventricular hemorrhage (IVH) was seen in 94/260 infants (36.2%) with grade 3 IVH in 21 (8.1%) and grade 4 in 31 (11.9%). Ten infants did not meet criteria for head ultrasound due to higher birthweight (>1500 g) and gestational age (>32 weeks). Screening for retinopathy of prematurity (ROP) was performed among preterm infants <30 weeks gestation or <1500 g birth weight. Fifty-nine infants did not have ROP and 37 had stage 3 and 2 had stage 4 ROP. Five infants required Laser therapy (Fig. 1).

### Cost of iNO

The total cost of iNO therapy to KPSC for these 270 infants was $3,781,332. A total of 39,804 h of iNO use occurred in the NICU during the study period among infants <34 weeks gestation. The median cost per patient was $7695 with an interquartile range of $9975.

### Survival to discharge and NICU mortality

Survival to discharge overall was 63% (169/270). Nine infants were transferred to level 4 NICUs outside the KPSC network. Survival to discharge among infants who had rupture of membranes (ROM) at delivery was 62.7% (116/185), 71.4% (10/14) with ROM of 1–18 h, 78.3% (18/23) with ROM of 19–120 h and 52.1% with PROM > 120 h (25/48). The overall survival to discharge among infants with ≤18 h of ROM was 63.3% (126/199) and was not statistically different from the 60.6% (43/71) survival among infants with ROM > 18 h. Twenty-eight infants exposed to iNO had a clinical diagnosis of pulmonary hypoplasia and 14 (50%) died. Survival among male infants was 99/154 (64%) compared to 70/116 (60%) among female infants. When adjusted for gestational age, AGA/SGA/LGA status and facility, female sex [OR 2.69 (1.19–7.41), p – 0.02] was associated with improved survival.

### Echocardiographic evidence of PH and survival

Survival to discharge was not different among infants with echocardiographic evidence of PH anytime either before or after initiation of iNO (71.9%–61/86) when compared to those who had no PH on echocardiogram (65.9%–85/129). However, many of these echocardiograms were performed a few days prior to or after initiation of iNO. We selected 58 infants who had echocardiography performed within 24 h prior to initiation of iNO. Among these infants, presence of echocardiographic evidence of PH associated with initiation of iNO during the first 10 postnatal days was associated with improved survival based on unadjusted analysis (Fig. 2B). However, when analysis was adjusted for gestational age, sex, AGA/SGA/LGA status and facility, there was no significant association between presence of PH on an echocardiogram and survival with OR 1.146 (0.45–2.93, p-0.78).

### Severity of HRF and survival

Severity of HRF was related to mortality. Mortality was lower when oxygenation index (OI) was <20 at initiation of iNO (24%) as compared to an OI of 21–40 (45%) or >40 (56%). Since arterial access was not always available at the time of initiation of iNO, we assessed severity of HRF using oxygen saturation index (OSI – Supplementary Table 1). High OSI was associated with increased mortality.

*Timing of initiation of iNO* had an impact on NICU mortality. Initiation of iNO in the first 72 h after birth was associated with a mortality rate of 44% (64/145). Initiation of iNO in the first week (33.8%–53/157) or 1–4 weeks (21.3%–16/75) was associated with lower mortality compared to initiation of iNO after 4 weeks of postnatal age (84%–32/38).

### Effect of race and ethnicity on mortality (Table 1)

The NICU mortality in Hispanic infants was 38.2% (49/128), 42% among White (26/62) and 26.5% among Black infants (13/49). This difference was not statistically significant.

### Effect of sex (Supplementary Table 3)

There was no sex difference in mortality. Differences in mortality among male and female among Black (20.7 vs. 35% respectively), Hispanic (39 vs. 37%) or White infants (37 vs. 50%) did not reach statistical significance.

### Growth status (AGA/SGA/LGA)

Mortality among small for gestational age infants (SGA) was 27% (10/37), appropriate for gestation (AGA) was 39% (83/212) and among large for gestational age (LGA) infants was 42% (9/21).

The status at discharge from the NICU is shown in Table 3. Seventy-nine infants were discharged home in room air, 71 infants were discharged on supplemental oxygen through a nasal cannula and 19 infants needed a tracheostomy.

**Table 3:** Outcomes at NICU discharge.

Outcome	*N*	Denominator	%
Mortality to NICU discharge (all infants <34 wks. *N* = 270)	101	270	37.4%
Among infants with gestational age < 30 weeks *n* = 221			
Mortality <36 wk. PMA	77	221	34.8%
BPD (VON definition) among survivors	98	138	71.0%
BPD grade 1–2 (Jensen)	85	132	64.4%
BPD grade 3 (Jensen)	27	125	21.6%
Among all infants < 34 weeks at discharge			
Supplemental oxygen by nasal cannula	71	169	42%
Tracheostomy and supplemental oxygen	19	169	11.2%
Home ventilator of those discharged home	9	152	5.9%
IVH grade ≥3 of those with ultrasound	52	260	20.0%
ROP stage ≥3 in eligible patients	39	168	23.2%
Medications at discharge			
Sildenafil - oral	11	169	6.5%
Albuterol - inhaled	38	169	22.5%
Budesonide – inhaled	55	169	32.5%
Furosemide – oral	9	169	5.3%
Hydrochlorothiazide – oral	25	169	14.8%
Spironolactone – oral	13	169	7.7%
Hydrochlorothiazide + spironolactone - oral	17	169	10.1%
Caffeine - oral	15	169	8.9%
Prednisolone or hydrocortisone – oral	6	169	3.6%

### Follow-up at 12 months

Sixty-three percent (169/270) of preterm infants who received iNO were discharged alive from the NICU including 9 transfers. Three infants transferred for higher level of care died and 3 were discharged on home ventilator.

Five infants died after NICU discharge at 4 months, 6 months, 1, 2, and 3 years of age. Follow-up information was available for 152 infants (90% of discharged patients). Overall, 19 infants required tracheostomy and 9 were discharged on home ventilation.

The weight, length, and head circumference of all 152 infants were compared using Z-scores at birth, NICU discharge, and at 12 months follow-up using Fenton and WHO growth curves. There was a significant decrease in the Z-score for all three growth parameters from birth to discharge (supplementary Fig. 1). There was a further decrease in length between discharge and 12 months. There was no statistically significant difference between Z-scores for weight and head circumference after discharge.

Mortality after discharge, rehospitalization before 12 months of age, medication use, supplemental oxygen needs, and echocardiographic findings at follow-up are shown in supplementary Table 2. Sixteen infants needed sildenafil therapy and only 4 demonstrated PH on echocardiogram at 12 months. Of note, two infants developed hepatoblastoma and one of them died at 2 years of age. The second infant with hepatoblastoma received chemotherapy and survived. Baseline demographics, characteristics of iNO use and outcomes based on sex are shown in supplementary Table 3.

## Discussion

We present a large case series of 270 preterm infants treated with iNO for indications not approved by the FDA, in a healthcare system with close follow-up. That the rate of iNO exposure in our population is similar to that in the California Perinatal Quality Care Collaborative during the same period suggests that our use of iNO is similar to that in California as a whole.

The most common indication for iNO was HRF (73.7%) not corrected by ventilation with 100% oxygen mostly during the first postnatal week (58.1%). Mortality was high (37.4% by NICU discharge) with 34.9% risk of rehospitalizations among survivors. Presence of PH, PROM or diagnosis of pulmonary hypoplasia was not associated with improved survival following iNO therapy. However, 105 (39% of all exposed to iNO and nearly two-thirds of survivors) infants were in room air, 41 (15.2%) were not on any medications and only 4 had evidence of PH at 12 months follow-up.

The use of iNO in preterm infants has not decreased in US following the publications of guidelines and clinical reports by the NIH and AAP and has increased especially among extremely preterm infants in UK [5, 23, 24]. Substantial variation in iNO use is observed between centers due to lack of clear guidelines. Guidelines from AHA/ATS suggest selective use of iNO in preterm infants with PH physiology or oligohydramnios [15]. In our study, echocardiographic evidence of PH prior to initiation of iNO was not associated with improved survival when adjusted for gestational age, sex, growth status, and facility although unadjusted analysis suggested better survival in the presence of PH. Our data are limited due to low number (*n* = 58) of infants with an echocardiogram <24 h prior to initiation of iNO. In a recent preterm registry, presence of echocardiographic evidence of PH or PROM did not impact mortality in response to PH-targeted therapy such as iNO [25]. Mortality with PH-targeted therapy was 38% compared to 33% without PH-targeted therapy in this registry. Despite these findings, it may be prudent that an echocardiogram prior to initiation of iNO in a preterm infant with hypoxemia during the first 10 days needs to be considered. The frequency of echocardiograms prior to iNO after the first 10 postnatal days was low in our study preventing us from making generalized recommendations. We speculate that other etiologies of hypoxemia such as parenchymal lung disease including severe RDS, and BPD does not respond well to iNO in the absence of PH.

Another interesting finding in our study is that timing of iNO was not associated with echocardiography. Although it is difficult to draw definitive conclusions from this finding, it indirectly suggests that hypoxic respiratory failure with high inspired oxygen need was the primary indication for iNO and not echocardiographic evidence of PH.

Contrary to our hypothesis, the survival among infants with PROM was not different from infants those who had ROM at birth. The diagnosis of pulmonary hypoplasia also was not associated with increased survival. These results contradict limited data from one randomized trial with 12 infants with pulmonary hypoplasia [12]. In a post-hoc analysis of a large randomized controlled trial [26], iNO reduced mortality (67% in the control group and 33% in the iNO group). A large observational study evaluated preterm infants with pulmonary hypoplasia [11]. In the study from the Pediatrix® database, mortality was almost a third lower in infants with PH and pulmonary hypoplasia exposed to iNO but this difference narrowly missed statistical significance [27]. Dyess et al reported a tendency towards lower mortality with PH-targeted therapy in the presence of PH+pulmonary hypoplasia (41%) compared to without PH-targeted therapy (60%) [25]. However, the number of infants with PH and pulmonary hypoplasia was small (*n* = 5). These variabilities in response to iNO and other PH therapies associated with PROM, oligohydramnios and pulmonary hypoplasia could be secondary to lack of a standard definition for pulmonary hypoplasia among preterm infants.

The growth of these preterm infants decreased during their NICU stay. However, such extrauterine growth restriction is commonly described in all preterm infants [28]. Length continued to decrease after discharge in these infants (supplementary Fig. 1) [29]. These findings emphasize the need for close nutritional assessment during follow-up of these infants.

The occurrence of two cases of hepatoblastoma (2/270 = 0.7% of all infants or 2/159 = 1.3% of survivors) is similar to childhood malignancy incidence of 1% reported by Dixon et al among neonates exposed to iNO [30, 31]. The precise role of iNO in triggering these malignancies is not clear as other factors such as radiation, phototherapy, medications and oxygen may potentially play a role [31].

There are several limitations to our study. Being an observational study limited to infants receiving iNO, we cannot ascertain the overall impact of iNO on the outcomes observed in these infants. We chose not to include a non-iNO control group because the observed challenges of identifying meaningfully matched controls when so many important variables such as ventilator management practices and compliance with iNO initiation protocols are difficult to characterize using retrospective data from the medical record. Such difficulties have been observed in other iNO studies [5]. It is somewhat reassuring that the rate of iNO use in our study population was similar to that in California as a whole. We also did not have a specific protocol for initiation and weaning of iNO in these units. Lack of follow-up data on babies transferred to level 4 NICUs outside KPSC is a source of bias. The strengths of the study include inclusion of all infants exposed to iNO with accurate data on initiation and duration of iNO and longitudinal follow-up. With the lack of equipoise to conduct a large randomized controlled trial of iNO in preterm infants, evaluation of data from large observational studies such as ours may currently be the best evidence to evaluate the effectiveness of iNO.

In conclusion, data from our regional health care system shows that preterm infants treated with iNO had relatively high NICU mortality and readmission rates after NICU discharge though two-thirds of the NICU survivors were in room air without any evidence of PH by one year of age. Inhaled NO treatment was primarily associated with HRF rather than PH. In our cohort, pulmonary hypertension (PH) was not associated with risk-adjusted 2 h oxygenation response or improved survival. This suggests that iNO therapy may be an indicator of disease severity rather than an indicator of a process where clinically meaningful outcomes can be modified by iNO.

## Summary

### What’s Known on This Subject

- Inhaled nitric oxide (iNO) improves survival without ECMO in term infants with hypoxemic respiratory failure. Randomized trials have not shown a mortality benefit with iNO in preterm infants. However, iNO is commonly used, especially in the first postnatal week in preterm infants.

### What This Study Adds

- In a retrospective study, 2.6% of preterm infants <34 weeks were exposed to iNO secondary to hypoxemia with high FiO_2_ requirement at initiation. These infants had 37% mortality prior to discharge and high 12-month respiratory morbidity. The presence of pulmonary hypertension at iNO initiation was not associated with improvement in acute oxygenation or in survival. This suggests that inhaled nitric oxide use in current practice may be an indicator of respiratory disease rather than mediator of improved outcomes.

## Supplementary information

Supplemental (online) figure 1

Supplemental (online) table 1

Supplemental (online) table 2

Supplemental (online) table 3

## Data Availability

The datasets generated during and analyzed during the current study are not publicly available due to HIPAA and privacy restrictions but could be made available from the corresponding author on reasonable request.
