# Supplementary material for: Retrospective study of preterm infants exposed to inhaled nitric oxide in Kaiser Permanente Southern California: morbidity, mortality and follow-up
Source: J Perinatol. 2024 Jul 18;45(4):506–12. doi: 10.1038/s41372-024-02051-w (PMC12069078; doi:10.1038/s41372-024-02051-w)
Supplement: Supplementary file 1 — Supplemental (online) figure 1 [file 41372_2024_2051_MOESM1_ESM.pptx]

## Slide 1
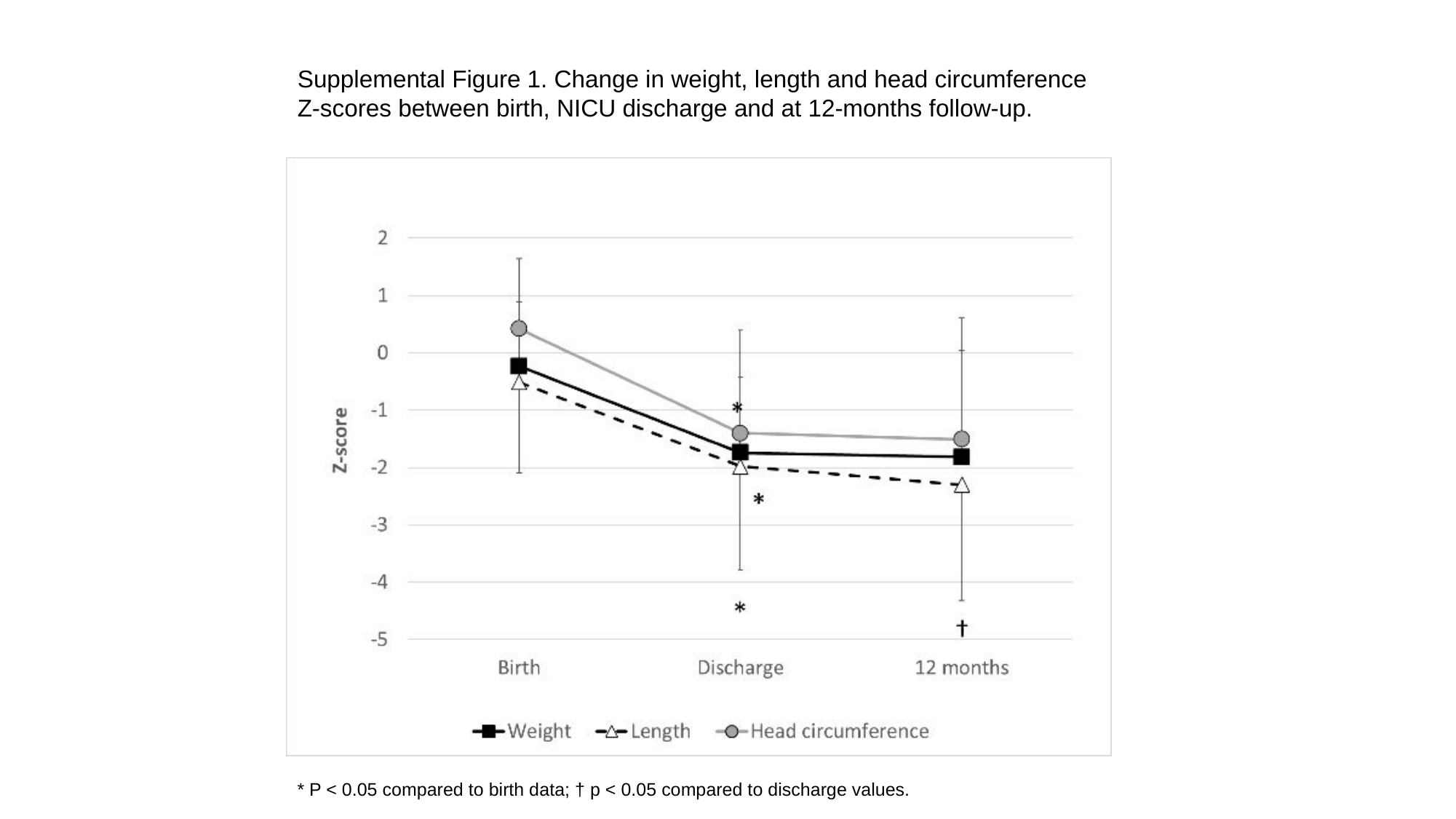

# Supplemental Figure 1. Change in weight, length and head circumference Z-scores between birth, NICU discharge and at 12-months follow-up.
* P < 0.05 compared to birth data; † p < 0.05 compared to discharge values.
