# Supplementary material for: Retrospective study of preterm infants exposed to inhaled nitric oxide in Kaiser Permanente Southern California: morbidity, mortality and follow-up
Source: J Perinatol. 2024 Jul 18;45(4):506–12. doi: 10.1038/s41372-024-02051-w (PMC12069078; doi:10.1038/s41372-024-02051-w)
Supplement: Supplementary file 2 — Supplemental (online) table 1 [file 41372_2024_2051_MOESM2_ESM.docx]

| **Supplemental (online) Table 1. Oxygen saturation index (OSI) at initiation of iNO and mortality** | | | |
| --- | --- | --- | --- |
| OSI Group | Total Cases | Deaths | Mortality (%) |
| 0-5 | 6 | 1 | 17 |
| 5.1-10 | 25 | 3 | 12 |
| 10.1-15 | 73 | 27 | 37 |
| > 15 | 157 | 66 | 42 |
| No OSI (on BMV) | 4 | 4 | 100 |
| No SpO_2_ or mean airway pressure recorded | 5 | 0 |  |
| Total | 270 | 101 | 37 |

 BMV – bag mask ventilation as infants were not tolerating being on a ventilator
