# Supplementary material for: Retrospective study of preterm infants exposed to inhaled nitric oxide in Kaiser Permanente Southern California: morbidity, mortality and follow-up
Source: J Perinatol. 2024 Jul 18;45(4):506–12. doi: 10.1038/s41372-024-02051-w (PMC12069078; doi:10.1038/s41372-024-02051-w)
Supplement: Supplementary file 3 — Supplemental (online) table 2 [file 41372_2024_2051_MOESM3_ESM.docx]

**Supplemental Table 2. Outcomes at 12 months* follow-up (as a percentage of infants with available follow-up data)**

| Outcome | N | % |  |
| --- | --- | --- | --- |
| Follow-up data available | 152/169 | 89.9% |  |
| Death after NICU discharge | 5 | 2.6% |  |
| Malignancy after NICU discharge | 2 (hepatoblastoma – 1 died at 2y) |  |  |
| No medications (inhaled or enteral) | 41 | 27.5% |  |
| Supplemental oxygen | 47 | 30.9% |  |
| Sildenafil | 16 | 10.5% |  |
| Bronchodilators | 103 (99-albuterol; 4-ipratropium) | 68% |  |
| Steroids (oral or inhaled) | 105 (56-budesonide; 8-fluticazone; 41-prednisolone (during 1^st^ year); | 69.1% |  |
| Diuretics | 40 (17-furosemide; 12-spironolactone; 11-hydrochlorthiazide + spironolactone) | 26.3% |  |
| Ped pulmonary consults | 73 | 48.0% |  |
| Echocardiograms | 35 | 23.0% |  |
| Pulmonary hypertension by echocardiogram. | 4 | 2.6% |  |
| Discharge to 12-months – Rehospitalizations infants | 53 | 34.9% |  |
| Multiple rehospitalizations during first 12 months | 14 (7 infants-twice; 4-thrice; 2-four times; one infant-5 times) | | |
| Discharge to 12-month total rehospitalizations | 78; 25-respiratory; 53-non-respiratory (hernia repair, shunt, ROP, G-tube etc.,) | | |

*Some information, such as onset of hepatoblastoma, occurred after 12 months of age but were included here for clinical relevance
