# Supplementary material for: Retrospective study of preterm infants exposed to inhaled nitric oxide in Kaiser Permanente Southern California: morbidity, mortality and follow-up
Source: J Perinatol. 2024 Jul 18;45(4):506–12. doi: 10.1038/s41372-024-02051-w (PMC12069078; doi:10.1038/s41372-024-02051-w)
Supplement: Supplementary file 4 — Supplemental (online) table 3 [file 41372_2024_2051_MOESM4_ESM.docx]

**Supplemental table 3. Characteristics of preterm infants exposed to iNO (n = 270) based on sex**

| Characteristics | Female n (%) | Male n (%) |
| --- | --- | --- |
| Delivery by cesarean section | 83 (41.5%) | 117 (58.5%) |
| Gestational age in weeks (median, range) | 116 (26.0, 22 5/7 – 33 5/7) | 154 (26.1, 22 5/7 – 33 5/7) |
| Birth weight in grams (median, range) | 772.5 (270-3195) | 790 (390-3200) |
| Small for gestational age (SGA) | 11 (29.7%) | 26 (70.3%) |
| Ethnicity and Race (by maternal self-report) |  |  |
| Hispanic (%) | 59 (46%) | 69 (54%) |
| White (%) | 24 (39%) | 38 (61%) |
| Black or African American (%) | 20 (41%) | 29 (59%) |
| Asian (%) | 11 (46%) | 13 (54%) |
| Hawaiian (%) | 2 (67%) | 1 (33%) |
| Apgar score – 1 min (median, range) | (Total=115) 4 (0-9) | (Total=154) 4 (0-9) |
| Apgar score – 5 min (median, range) | (Total=116) 7 (1-9) | (Total=154) 7 (1-9) |
| Age at iNO initiation (h) median IQR | (Total=116) 141 (1-3582) | (Total=154) 129 (1-3600) |
| <8 Days (Total=157) | Total=66 (42%) | Total=91 (58%) |
| 8-28 Days (Total=75) | Total=33 (44%) | Total=42 (56%) |
| >28 Days (Total=38) | Total=17 (45%) | Total=21 (55%) |
| Pre-INO respiratory support |  |  |
| Surfactant (Total=262) | Total=112 (43%) | Total=150 (57%) |
| Non-invasive positive pressure ventilation (Total=2) | Total=0 (0%) | Total=2 (100%) |
| Invasive mechanical ventilation (Total=19) | Total=10 (53%) | Total=9 (47%) |
| High frequency oscillator (Total=239) | Total=104 (44%) | Total=135 (56%) |
| High frequency jet ventilator (Total=7) | Total=1 (14%) | Total=6 (86%) |
| PPV (bagging or T-piece) (Total=3) | Total=1 (33%) | Total=2 (67%) |
| Pre-iNO OI† (data from 176 infants with arterial access) (Total=176) | Total=74 (42%) | Total=102 (58%) |
| Mild HRF OI ≤ 15 (%) (Total=25) | Total=7 (28%) | Total=18 (72%) |
| Moderate HRF OI 16-25 (%) (Total=39) | Total=17 (44%) | Total=22 (56%) |
| Severe HRF OI 26-40 (%) (Total=66) | Total=33 (50%) | Total=33(50%) |
| Critical HRF OI > 40 (%) (Total=46) | Total=17 (37%) | Total=29 (63%) |
| Pre-iNO OSI# (median – IQR) (Total=89) BVM+PPV=5 cases excluded (94-5=89 cases) | Total=40 (45%) 18 (2-73) | Total=49 (55%) 18 (2-73) |
| Pulmonary hypertension on echocardiogram (Total=88) | Total=38 (43%) | Total=50 (57%) |
| iNO dose, initial (ppm) – median and range (Total=270) | Total=116 (43%) 20 (5-40) | Total=154 (57%) 20 (5-20) |
| iNO duration (h) median IQR (Total=270) | Total=116 (43%) 76 (1-2564) | Total=154 (57%) 76 (1-1178) |
| Mortality to NICU discharge (all infants < 34 wks. N = 270) (Total Died=101) | Total=46 (36%) | Total=55 (54%) |
| Among infants with gestational age < 30 weeks n = 221 (Tolal Died=89) | Total=52 (47%) | Total=47 (53%) |
| BPD (VON definition) among survivors (Total=97) | Total=42 (43%) | Total=55 (57%) |
| Tracheostomy and supplemental oxygen (Total=18) | 9 (50%) | 9 (50%) |
| Home ventilator of those discharged home (Total=9) | 5 (56%) | 4 (44%) |
| Sildenafil (Total=16) | 9 (56%) | 7 (44%) |
